# Management of pregnancy in women with monogenic diabetes due to mutations in *GCK*, *HNF1A* and *HNF4A* genes

**DOI:** 10.3389/fgene.2024.1362977

**Published:** 2024-06-12

**Authors:** M. T. Crowley, B. Paponette, S. Bacon, M. M. Byrne

**Affiliations:** ^1^ Department of Endocrinology and Diabetes, Mater Misericordiae University Hospital, Dublin, Ireland; ^2^ Department of Endocrinology and Diabetes, Sligo University Hospital, Sligo, Ireland

**Keywords:** MODY, pregnancy, *HNF1A*, *HNF4A*, *GCK*, macrosomia, neonatal hypoglycaemia

## Abstract

Women with maturity-onset diabetes of the young (MODY) need tailored antenatal care and monitoring of their offspring. Each MODY subtype has different implications for glycaemic targets, treatment choices and neonatal management. Hyperglycaemia of MODY is often first diagnosed in adolescence or early adulthood and therefore is clinically relevant to pregnant women. MODY remains an under-recognised and undiagnosed condition. Pregnancy represents an opportune time to make a genetic diagnosis of MODY and provide precision treatment. This review describes the nuance of antenatal care in women with MODY and the implications for pregnancies affected by a positive paternal genotype. Mutations in hepatic nuclear factor 1-alpha (*HNF1A*) and 4-alpha (*HNF4A*) genes are associated with progressive β-cell dysfunction resulting in early onset diabetes. Patients are largely managed with sulphonylureas outside of pregnancy. Macrosomia and persistent neonatal hypoglycaemia are reported in 54% and 15% of *HNF4A* genotype positive offspring respectively with a median increase in birthweight of 790 g. Close observation of foetal growth *in utero* allows optimal timing of delivery to minimise peri- and postpartum materno-foetal complications. Glucokinase (*GCK*)-MODY causes mild fasting hyperglycaemia which does not require treatment outside of pregnancy. Birthweight of offspring of maternal carriers is dependent on foetal genotype; heterozygous mutation carriers are usually normal weight while genotype negative offspring are large for gestational age (600 g heavier). Affected offspring of paternal carriers may be small for gestational age (500 g lighter). Serial growth scans with measurement of the abdominal circumference indirectly differentiate foetal genotype. Measurement of cell free foetal DNA in maternal blood from the late first trimester is superior to traditionally used ultrasound to distinguish foetal genotype. Cost and accessibility may limit its use.

## 1 Introduction

MODY is a phenotypically and genetically heterogenous subtype of monogenic diabetes defined as young onset of non-insulin dependent diabetes with a pattern of dominant inheritance. The most common forms of MODY in adults include GCK-MODY (30%–50%) and HNF1A-MODY (30%–50%) while HNF4A-MODY is much rarer (2%–5%) (Shields et al., 2010; Kyithar et al., 2011). Up to one in 1500 individuals carry a pathogenic variant for one of these MODY subtypes (Mirshahi et al., 2022). All of these mutations exhibit autosomal dominant inheritance. Both paternal and maternal inheritance can affect foetal development *in utero*, as well as perinatal and neonatal outcomes. These clinical implications can often be under recognised as MODY remains frequently undiagnosed or misdiagnosed as type 1, type 2 or gestational diabetes (GDM).

## 2 Principles of management of diabetes in pregnancy

Pregestational diabetes is associated with increased incidence of embryopathy directly proportional to HbA_1c_ elevation in early pregnancy (Guerin et al., 2007). Rigorous pre-pregnancy planning facilitates commencement of high dose folic acid, ensures up to date retinal screening, discontinuation of potential teratogenic medication and optimsation of dysglycaemia to a HbA_1c_ of less than 42 mmol/mol (ElSayed et al., 2022).

GDM occurs exclusively in pregnancy. Some deliberation exists around the diagnostic thresholds for GDM. The International Association of Diabetes in Pregnancy Study Group criteria are when one or more values equals or exceeds thresholds of a fasting glucose of 5.1 mmol/L, 1-h of 10 mmol/L or 2 h of 8.5 mmol/L following a 75 g oral glucose tolerance test preceded by an overnight fast (Metzger et al., 2010). Suboptimal glycaemic control is associated with macrosomia and its inherent morbidity at delivery and in the neonatal period. Therefore, the American Diabetes Association endorse stringent pregnancy targets for women with GDM of fasting glucose of less than 5.3 mmol/L and 1 h post-prandial value of less than 7.8 mmol/L (ElSayed et al., 2022).

Insulin is considered the gold standard treatment option for women with diabetes in pregnancy despite being labour intensive for women in administration and the inherent risk of hypoglycaemia (ElSayed et al., 2022). Metformin use has historically been linked to small for gestational age infants (Rowan et al., 2008; Feig et al., 2020), although more recent data disputes this and reinforces that metformin is associated with less maternal weight gain, less insulin requirement and less incidence of neonatal macrosomia (Dunne et al., 2023). Sulphonylurea (SU) drugs, such as glibenclamide, undergo placental-foetal transfer, thereby increasing foetal size and incidence of neonatal hypoglycaemia (Camelo Castillo et al., 2015). One meta-analysis observed a relative risk ratio of macrosomia and neonatal hypoglycaemia of 2.62 (95% CI 1.35–5.08) and 2.04 (95% CI 1.30–3.20) respectively in offspring where women with GDM were treated with SU (Balsells et al., 2015). As a result of these concerns, SU agents are not routinely used in pregnancy.

## 3 HNF1A-MODY

HNF1A-MODY accounts for approximately 30%–50% of MODY cases (Shields et al., 2010; Kyithar et al., 2011). The estimated prevalence of *HNF1A* mutations in women affected by GDM is less than 1% (Weng et al., 2002; Zurawek et al., 2007). Numerous cases in published retrospective reviews are diagnosed at a later age due to familial screening once generational inheritance has been established.

### 3.1 Pathophysiology

HNF1A-MODY is due to an inactivating germline mutation in the gene encoding HNF1α in chromosome 12q identified in 1995 (Menzel et al., 1995; Vaxillaire et al., 1995; Yamagata et al., 1996a). HNF1α is a transcription factor expressed in the pancreas, liver (Odom et al., 2004), kidney (Pontoglio et al., 2000) and gut (Lussier et al., 2010). Within the pancreatic β-cell, HNF1α acts as a regulator of insulin secretion in part through influence of glucose transporter 2 (GLUT2) (Luni et al., 2012). Inactivating mutations of *HNF1A* result in impaired insulin secretion in β-cells with decreased insulin response to high glucose evident in genotype positive prediabetic subjects (Byrne et al., 1996).

Affected individuals are typically young and lean with a progressive β-cell defect and variable treatment requirements over time. Fasting glucose levels are often mildly raised while post-prandial values show a relatively high increment. Diabetes exposure *in utero* results in an earlier age at diagnosis of diabetes in heterozygous *HNF1A* offspring (15.5 ± 5.4 vs. 27.5 ± 13.1 years, *p* = 0.05) (Stride et al., 2002).

Glucosuria is seen at relatively normal glucose values due to altered renal glucose reabsorption (Menzel et al., 1998). Liver adenomatosis has been observed in some cases (Reznik et al., 2004). First line treatment outside of pregnancy is with low dose SU which bind to the SUR1 subunit and close the ATP-sensitive potassium channel to stimulate insulin release from the β-cell, bypassing the effects of *HNF1A* on the GLUT2 pathway (Pearson et al., 2003; Bacon et al., 2016a). Glucagon-like peptide 1 agonist therapy could potentially be used as a second line treatment option for this cohort (Østoft et al. 2014).

### 3.2 Characteristics of HNF1A-MODY in pregnancy

To date, no significant difference in birth weight and rates of macrosomia has been shown in neonates inheriting a *HNF1A* mutation when compared to unaffected siblings (Pearson et al., 2007; Bacon et al., 2015). One retrospective review showed that birthweight among 85 heterozygous *HNF1A* offspring was similar to 49 familial controls who did not carry the *HNF1A* mutation (median difference 10 g, *p* = 0.86) (Pearson et al., 2007). Another cohort study showed a median birthweight was 3.6 kg (IQR 3.1–4.0 kg) corresponding to the 60th centile when corrected for gestational age (Bacon et al., 2015). Incidence of macrosomia and prolonged hypoglycaemia were noted to be higher in offspring of non-insulin treated pregnancies although this did not reach statistical significance.

Diazoxide-responsive congenital hyperinsulinaemic hypoglycaemia (CHI) is very rare but has been described in offspring with *HNF1A* mutations (Brusgaard et al., 2006; Dusatkova et al., 2011; Stanescu et al., 2012). A retrospective study from a large paediatric unit showed that 3.4% of CHI cases were due to *HNF1A* mutations (Tung et al., 2018). Median birthweight was 3815 g (IQR 3317–4176 g) and the majority of cases were paternally inherited. The age of presentation with hypoglycaemia varied from one day to three months old with diazoxide treatment requirement up to 7.3 years.

### 3.3 Management of HNF1A-MODY in pregnancy

#### 3.3.1 Diabetes care

Outside of pregnancy, HNF1A-MODY is optimally managed with low dose SU therapy (Pearson et al., 2003; Bacon et al., 2016a). Management of dysglycaemia in pregnancy is nuanced as outlined in Table 1. SU use has not been studied in MODY affected pregnancies. Insulin remains the first line therapy during pregnancy. Initiation pre-pregnancy allows for cessation of SU and optimisation of glycaemic control. Insulin therapy was initiated in 26% of pregnancies in the only study describing glycaemic treatment (Bacon et al., 2015). Insulin dose requirements were 0.4unit/kg/day (0.2–0.60) in the first trimester and increased to 0.8unit/kg/day (0.6–0.9) in the third trimester.

**TABLE 1:** Suggested management of HNF1A- and HNF4A-MODY in pregnancy according to parental history.

Parental carrier	Pre-conception	Second trimester	Third trimester	Post-delivery
Maternal	Optimisation of glycaemic control^a^	Switch to insulin if on SU	Monitor for increased foetal size, particularly if HNF4A-MODY	Monitor for neonatal hypoglycaemia for 48 h following delivery, particularly if HNF4A-MODY
	Option 1: transition from SU to insulin			
	Option 2: switch from alternative SU to glibenclamide		Consider early delivery if foetal size increased	
Paternal	Alert patient and obstetrician to features of HNF1A- or HNF4A-MODY in pregnancy			

^a^ Dependent on current treatment, HbA_1c_ and patient preference.

A switch from SU treatment to insulin in the first trimester may transiently compromise glycaemic control with potential effect on organogenesis. Treatment guidelines suggest that glibenclamide can be continued until the second trimester of pregnancy provided that glycaemic targets for pregnancy including a HbA_1c_ of less than 42 mmol/mol are achieved (Shepherd et al., 2017). No increased incidence of congenital anomalies or pregnancy loss has been observed in first trimester use of SU (Towner et al., 1995). Therefore, the decision on switch from SU in early pregnancy is based on maternal glycaemic control, type and dosage of SU agent at the time of conception and patient choice.

#### 3.3.2 Obstetric and postpartum care

Regular foetal growth assessment from 28 weeks gestation in line with guidelines for pre-GDM aids decision making on timing of delivery (Shepherd et al., 2017). Delivery at 37 to 38^+6^ weeks should be considered according to foetal growth.

Glibenclamide is not secreted in breastmilk or associated with neonatal hypoglycaemia at doses of 5–10 mg daily (Feig et al., 2005). Gliclazide, a more commonly available agent, has not been studied. Therefore, women who breastfeed with persistent dysglycaemia postpartum may switch to glibenclamide, provided dose requirements are relatively modest, or continue on insulin treatment.

Neonatal monitoring for hypoglycaemia should be performed following delivery. Offspring of *HNF1A* heterozygous carriers who develop CHI without a history of perinatal stress should be screened for *HNF1A* gene mutations (Tung et al., 2018).

## 4 HNF4A-MODY

The genetic basis of HNF4A-MODY was first described in 1996 (Yamagata et al., 1996b). Phenotypic features of young onset of diabetes in a lean individual with predominant postprandial dysglycaemia overlaps with HNF1A-MODY. Foetal hyperinsulinism, macrosomia and CHI complete the dual phenotype and appear to be more common in HNF4A-MODY than HNF1A-MODY (Stanescu et al., 2012). Hyperinsulinism can persist into early adulthood (Bacon et al., 2016b). SU are most commonly used to treat hyperglycaemia outside of pregnancy (Shepherd et al., 2018).

### 4.1 Pathophysiology

The pathophysiology that mediates this dual phenotype is not well understood. Clinical studies show loss of *HNF4A* function leads to β-cell dysfunction and impaired insulin secretion (Byrne et al., 1995). Inactivation of the *HNF4A* gene in mice resulted in hyperinsulinaemia *in utero* and overt hypoglycaemia in early life (Gupta et al., 2005; Pearson et al., 2007). One of those studies also observed impaired glucose tolerance and 60% reduced expression of *KCNJ11* which encodes the Kir6.2 subunit of the potassium channel on the GLUT2 transporter (Gupta et al., 2005), although this was not replicated in another study where expression of Kir6.2 was normal (Pearson et al., 2007). One hypothesis proposed foetal insulin hypersecretion induces β-cell failure later in life resulting in diabetes. Another suggested that two separate *HNF4A* gene-expression defects could result in this contrasting biphasic phenotype (Pearson et al., 2007).

### 4.2 Characteristics of HNF4A-MODY in pregnancy

A significant increase in birthweight was seen in 54 *HNF4A* carriers compared to non-affected family members (median 790 g, *p* < 0.001) (Pearson et al., 2007). In the same group, the incidence of macrosomia was four times higher in *HNF4A* carriers compared to the non-mutation family members (56% vs. 13%, *p* < 0.001). This effect was heightened if the *HNF4A* mutation was maternally inherited (median corrected birth weight 4,840 g when mother affected vs. 4,170 g when father affected). Similar results were seen in a more recent study of 186 affected individuals (Locke et al., 2022). Higher birthweight in affected offspring is associated with reduced penetrance of diabetes in childhood and early adulthood (Locke et al., 2022).

Importantly 46% of *HNF4A* genotype positive offspring with paternal inheritance were macrosomic at birth (Pearson et al., 2007). This suggests macrosomia in affected offspring correlates with foetal genotype and foetal intra-uterine hyperglycaemia exposure has an additional effect.

One centre reported that *HNF4A* mutations are the third most common cause of diazoxide-responsive CHI accounting for 18.6% of cases with identifiable genetic aetiology (Flanagan et al., 2010). CHI generally develops within the first week of life with the majority presenting within the first two days of life (Flanagan et al., 2010; Tung et al., 2018; McGlacken-Byrne et al., 2022). Inactivating *HNF4A* mutations associated with CHI tend to arise from the 7–9 isoforms expressed in the P2 promotor (Kapoor et al., 2008). Transient hypoglycaemia which, occurs within the first three days of life, was observed in 15.4% of mutation carriers in one cohort however this may be underestimated based on retrospective nature of study design with patient recall of perinatal events (Pearson et al., 2007).

### 4.3 Management of HNF4A-MODY in pregnancy

#### 4.3.1 Management of diabetes

There is limited evidence to guide maternal glycaemia management in pregnancy in HNF4A-MODY. Similar to HNF1A-MODY, women are ideally established on insulin therapy pre-pregnancy. Alternatively, women can continue on a pregnancy safe SU (glibenclamide) in the first trimester of pregnancy with a switch to insulin therapy in the second trimester (Shepherd et al., 2017). This is thought to avoid the macrosomic effect of glibenclamide in late pregnancy. The impact of maternal glycaemic control on birth weight and neonatal hypoglycaemia in HNF4A-MODY is not described in the literature.

#### 4.3.2 Obstetric and *postpartum* care

Management of a parental history of HNF4A-MODY in pregnancy is summarised in Table 1. It is recommended that serial growth assessments are performed from 28 weeks’ gestation at two weekly intervals to detect developing macrosomia (Shepherd et al., 2017). Early delivery is generally indicated if the foetus appears genetically affected based on foetal size. Macrosomia confers an increased risk of shoulder dystocia, brachial plexus injury, prolonged second stage of labour, assisted delivery and need for emergency caesarean section (Kapoor et al., 2008).

Importantly, heterozygous *HNF4A* mutations of paternal inheritance are associated with macrosomia (Pearson et al., 2007). A paternal history of HNF4A-MODY warrants the same frequency of assessment of foetal growth *in utero*, i.e., serial growth assessments are performed from 28 weeks’ gestation at two weekly intervals to detect developing macrosomia (Shepherd et al., 2017).

Cell free DNA has been used to determine foetal genotype in GCK mutations and ABCC8 and may be beneficial in the future in others forms of MODY including HNF4A (De Franco et al., 2017; Hughes et al., 2023). Detection of a paternally inherited *HNF4A* mutation in the cell free foetal DNA in a maternal blood sample may help to stratify neonatal risk of macrosomia and CHI following delivery. Use of cell free DNA in cases of potential paternal inheritance may be considered in clinical practice in the near future although not reported in the literature yet.

Monitoring for neonatal hypoglycaemia is required for at least 48 h in the postpartum period. Neonatal hypoglycaemia, independent of maternal glycaemia, is seen in at least 10% of affected neonates with a proportion requiring prolonged treatment for up to a number of months (Shepherd et al., 2017).

Similar to HNF1A-MODY, women with persistent dysglycaemia following pregnancy may be able to switch to SU therapy and glibenclamide in particular if breastfeeding in the postpartum period.

## 5 GCK-MODY

### 5.1 Pathophysiology

GCK-MODY is due to a heterozygous inactivation of the glucokinase gene located on chromosome 7b (Froguel et al., 1992). Glucokinase acts as a glucose sensor in the pancreatic β-cell. Subjects with this genetic variation typically present with mild fasting plasma glucose (5.5–8 mmol/L), an increment of less than 4.6 mmol/L following glucose load and HbA_1c_ values less than 60 mmol/mol (7.6%) (Steele et al., 2013). Studies have shown that oral hypoglycaemic agents are not recommended and are ineffective in GCK-MODY patients. (Stride et al., 2014). Individuals are usually asymptomatic and do not develop long term micro and macrovascular complications that frequently occur in diabetes (Steele et al., 2014).

According to the Atlantic Diabetes in Pregnancy cohort study, population prevalence of GCK-MODY is 1.1 in 1000 (Chakera et al., 2014). Pregnancy is an opportunity for case detection as many women are screened for GDM. Fasting glucose greater than 5.5 mmol/L and BMI less than 25 kg/m^2^ confer a specificity of 98% and sensitivity of 68% for *GCK* diagnosis in this Caucasian population although may not be accurate for different cohorts (Chakera et al., 2014).

### 5.2 Characteristics of GCK-MODY in pregnancy

Foetal endogenous insulin secretion is influenced by maternal hyperglycaemia. A genetically unaffected foetus of a woman with a *GCK* mutation is 600 g heavier at birth with a higher risk of foetal macrosomia due to dysregulated insulin stimulated foetal growth (Hattersley et al., 1998; Spyer et al., 2009). To avoid this, it is recommended to normalise hyperglycaemia in maternal *GCK* carriers of an unaffected foetus through the use of insulin therapy (Hattersley et al., 1998; Chakera et al., 2015). In contrast, when the foetus inherits the maternal gene, foetal growth is normal due to a similar mildly elevated glucose set point *in utero*. Treatment with insulin in this scenario may precipitate foetal growth restriction (Timsit et al., 2021). Furthermore, birth weight is reduced by 500 g in paternally inherited foetal mutations where a mother is unaffected (Hattersley et al., 1998). The congenital malformation rate of GCK-MODY pregnant women’s offspring is 2.4% with no difference in genotype positive or negative offspring (Ren et al., 2023). There is a lower birth complication rate in genotype positive offspring compared to negative offspring.

### 5.3 Management of GCK-MODY in pregnancy

#### 5.3.1 Management of diabetes

**FIGURE 1:**
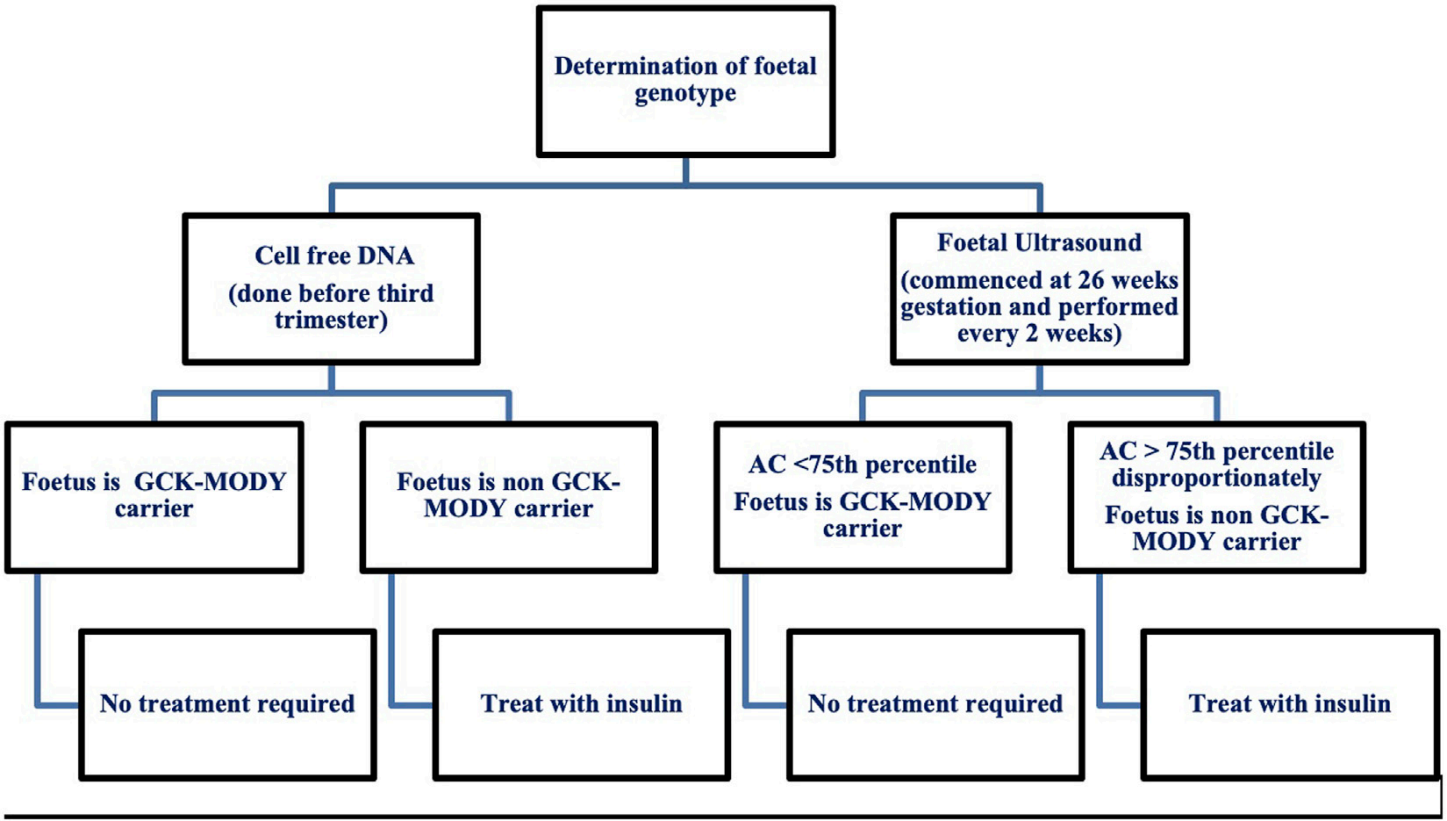
Current recommendations for the management of maternal GCK-MODY in pregnancy. AC, Abdominal Circumference.

Treatment in pregnancy poses a great clinical dilemma as insulin initiation is determined by foetal genetic inheritance (Chakera et al., 2012; Timsit et al., 2021). Current recommendations for the management of GCK-MODY in pregnancy are summarised in Figure 1. Once insulin is introduced, glucose targets for pregnancy can be elusive. A high prevalence of severe hypoglycaemia has been reported by *GCK* affected women treated with insulin (Dickens et al., 2019). Exogenous insulin treatment reduces endogenous insulin secretion as a consequence of defective glucose sensing in the β-cell. In addition, individuals have a higher hypoglycaemic threshold for counter-regulatory glucagon and epinephrine secretion which, potentially protects against hypoglycaemia (Guenat et al., 2000). Individuals with GCK-MODY experience autonomic symptoms at higher glucose levels making the traditional glycaemic targets for GDM difficult to achieve (Chakera et al., 2018).

#### 5.3.2 Obstetric and postpartum care

Invasive testing for determination of foetal genotype is not recommended due to the inherent risk of miscarriage. Foetal genotype is most commonly determined by assessment of foetal growth via serial ultrasound and more recently with use of cell free DNA. Serial ultrasounds are used in women with gestational diabetes where insulin treatment is titrated according to the acceleration of the foetal abdominal circumference (Timsit et al., 2021). Ultrasound in GCK-MODY pregnancies helps to classify foetal genotype by measurement of the abdominal circumference (AC) (Timsit et al., 2021). At 26 weeks gestation, an AC less than the 75th percentile suggests the foetus has inherited the *GCK* mutation and insulin is not recommended (Timsit et al., 2021).

An AC disproportionately greater than the 75th percentile suggests the foetus is a non-carrier and insulin is needed to prevent macrosomia and delivery should occur at 38 weeks (Timsit et al., 2021). Insulin initiation in the third trimester in this context has not been proven to prevent macrosomia. In one study *GCK* unaffected offspring whose mothers were treated with insulin had a lower rate of macrosomia compared to the non-insulin treated group (33.3% vs. 62.5%) (Bacon et al., 2015). Another study showed that insulin therapy in unaffected offspring did not reduce large for gestational age (López Tinoco et al., 2021).

A recent study has shown that assessment of foetal genotype via ultrasound is not accurate with a 53% sensitivity and 61% specificity for an AC greater than 75th percentile (Hughes et al., 2023). The use of cell free DNA is a promising diagnostic choice with a sensitivity of 100% and specificity of 96%. Results can be obtained prior to the third trimester which enables better pregnancy management with insulin (Hughes et al., 2023). Albeit an accurate diagnostic measure for foetal genotype classification, the biggest limiting factor for use of cell free DNA extraction is cost with an estimated cost of £2000 per patient (Verhoef et al., 2016; Hughes et al., 2023). With the upcoming use of cell free DNA, there is need for future studies to improve maternal management when foetal status is known (Hughes et al., 2023).

## 6 Conclusion

Pre-gestational diagnosis of MODY facilitates personalised antenatal treatment of women in pregnancy, foetal monitoring *in utero* and neonatal care after delivery. HNF4A- and HNF1A-MODY affected pregnancies require increased surveillance for foetal macrosomia *in utero* and neonatal hypoglycaemia soon after delivery. Numerous studies describe outcomes where women were managed according to local gestational diabetes guidelines and there is a paucity of literature comparing management of insulin and SU in this population. Glycaemic targets are modified in GCK-MODY affected pregnancies according to foetal genotype. The emerging evidence base for cell free DNA to determine foetal genotype in GCK-MODY affected pregnancies may aid clinical practice and have a broader application to antenatal care of other MODY subtypes.

## Abbreviations

GCK, Glucokinase; HNF4A, Hepatic nuclear factor 4-alpha; HNF1A, Hepatic nuclear factor 1-alpha; MODY, maturity onset diabetes of the young; GDM, gestational diabetes mellitus; SU, sulphonylurea; GLUT2, glucose transporter 2; CHI, congenital hyperinsulinaemic hypoglycaemia; AC, abdominal circumference.

## References

[B2] Bacon S. Kyithar M. P. Condron E. M. Vizzard N. Burke M. Byrne M. M. (2016b). Prolonged episodes of hypoglycaemia in HNF4A-MODY mutation carriers with IGT. Evidence of persistent hyperinsulinism into early adulthood. Acta Diabetol. 53 (6), 965–972. 10.1007/s00592-016-0890-9 27552834

[B3] Bacon S. Kyithar M. P. Rizvi S. R. Donnelly E. McCarthy A. Burke M. (2016a). Successful maintenance on sulphonylurea therapy and low diabetes complication rates in a HNF1A-MODY cohort. Diabet. Med. 33 (7), 976–984. 10.1111/dme.12992 26479152

[B4] Bacon S. Schmid J. McCarthy A. Edwards J. Fleming A. Kinsley B. (2015). The clinical management of hyperglycemia in pregnancy complicated by maturity-onset diabetes of the young. Am. J. Obstet. Gynecol. 213 (2), 236.e1–e7. 10.1016/j.ajog.2015.04.037 25935773

[B5] Balsells M. García-Patterson A. Solà I. Roqué M. Gich I. Corcoy R. (2015). Glibenclamide, metformin, and insulin for the treatment of gestational diabetes: a systematic review and meta-analysis. Bmj 350, h102. 10.1136/bmj.h102 25609400 PMC4301599

[B6] Brusgaard K. C. H. Hansen T. Njolstad P. Malec J. Brock J. B. (2006). A TCF1 mutation may cause transient congenital hyperinsulinism followed by MODY3. Endocr. Abstr. 11.

[B7] Byrne M. M. Sturis J. Fajans S. S. Ortiz F. J. Stoltz A. Stoffel M. (1995). Altered insulin secretory responses to glucose in subjects with a mutation in the MODY1 gene on chromosome 20. Diabetes 44 (6), 699–704. 10.2337/diab.44.6.699 7789636

[B8] Byrne M. M. Sturis J. Menzel S. Yamagata K. Fajans S. S. Dronsfield M. J. (1996). Altered insulin secretory responses to glucose in diabetic and nondiabetic subjects with mutations in the diabetes susceptibility gene MODY3 on chromosome 12. Diabetes 45 (11), 1503–1510. 10.2337/diab.45.11.1503 8866553

[B9] Camelo Castillo W. Boggess K. Stürmer T. Brookhart M. A. Benjamin D. K. Jr. Jonsson Funk M. (2015). Association of adverse pregnancy outcomes with glyburide vs insulin in women with gestational diabetes. JAMA Pediatr. 169 (5), 452–458. 10.1001/jamapediatrics.2015.74 25822253

[B10] Chakera A. J. Carleton V. L. Ellard S. Wong J. Yue D. K. Pinner J. (2012). Antenatal diagnosis of fetal genotype determines if maternal hyperglycemia due to a glucokinase mutation requires treatment. Diabetes Care 35 (9), 1832–1834. 10.2337/dc12-0151 22773699 PMC3425005

[B11] Chakera A. J. Spyer G. Vincent N. Ellard S. Hattersley A. T. Dunne F. P. (2014). The 0.1% of the population with glucokinase monogenic diabetes can be recognized by clinical characteristics in pregnancy: the Atlantic Diabetes in Pregnancy cohort. Diabetes Care 37 (5), 1230–1236. 10.2337/dc13-2248 24550216

[B12] Chakera A. J. Steele A. M. Gloyn A. L. Shepherd M. H. Shields B. Ellard S. (2015). Recognition and management of individuals with hyperglycemia because of a heterozygous glucokinase mutation. Diabetes Care 38 (7), 1383–1392. 10.2337/dc14-2769 26106223

[B64] Chakera A. J. Hurst P. S. Spyer G. Ogunnowo-Bada E. O. Marsh W. J. Riches C. H. (2018). Molecular reductions in glucokinase activity increase counter-regulatory responses to hypoglycemia in mice and humans with diabetes. Mol. Metab. 17, 17–27. 30146176 10.1016/j.molmet.2018.08.001 PMC6197723

[B13] De Franco E. Caswell R. Houghton J. A. Iotova V. Hattersley A. T. Ellard S. (2017). Analysis of cell-free fetal DNA for non-invasive prenatal diagnosis in a family with neonatal diabetes. Diabet. Med. 34 (4), 582–585. 10.1111/dme.13180 27477181 PMC5096683

[B14] Dickens L. T. Letourneau L. R. Sanyoura M. Greeley S. A. W. Philipson L. H. Naylor R. N. (2019). Management and pregnancy outcomes of women with GCK-MODY enrolled in the US Monogenic Diabetes Registry. Acta Diabetol. 56 (4), 405–411. 10.1007/s00592-018-1267-z 30535721 PMC6468988

[B15] Dunne F. P. Alvarez-Iglesias A. Newman C. Smyth A. Browne M. Devane D. (2023). 183-LB: a randomized placebo-controlled trial of the effectiveness of early metformin in addition to usual care in the reduction of gestational diabetes mellitus effects (emerge). Diabetes 72 (Suppl_1). 10.2337/db23-183-lb PMC9494837 36131291

[B16] Dusatkova P. Pruhova S. Sumnik Z. Kolouskova S. Obermannova B. Cinek O. (2011). HNF1A mutation presenting with fetal macrosomia and hypoglycemia in childhood prior to onset of overt diabetes. J. Pediatr. Endocrinol. Metab. 24 (3-4), 187–189. 10.1515/jpem.2011.083 21648289

[B17] ElSayed N. A. Aleppo G. Aroda V. R. Bannuru R. R. Brown F. M. Bruemmer D. (2022). 15. Management of diabetes in pregnancy: standards of care in diabetes—2023. Diabetes Care 46 (Suppl_1), S254–S266. 10.2337/dc23-S015 PMC9810465 36507645

[B18] Feig D. S. Briggs G. G. Kraemer J. M. Ambrose P. J. Moskovitz D. N. Nageotte M. (2005). Transfer of glyburide and glipizide into breast milk. Diabetes Care 28 (8), 1851–1855. 10.2337/diacare.28.8.1851 16043722

[B19] Feig D. S. Donovan L. E. Zinman B. Sanchez J. J. Asztalos E. Ryan E. A. (2020). Metformin in women with type 2 diabetes in pregnancy (MiTy): a multicentre, international, randomised, placebo-controlled trial. Lancet Diabetes Endocrinol. 8 (10), 834–844. 10.1016/S2213-8587(20)30310-7 32946820

[B20] Flanagan S. E. Kapoor R. R. Mali G. Cody D. Murphy N. Schwahn B. (2010). Diazoxide-responsive hyperinsulinemic hypoglycemia caused by HNF4A gene mutations. Eur. J. Endocrinol. 162 (5), 987–992. 10.1530/EJE-09-0861 20164212 PMC2857991

[B21] Froguel P. Vaxillaire M. Sun F. Velho G. Zouali H. Butel M. O. (1992). Close linkage of glucokinase locus on chromosome 7p to early-onset non-insulin-dependent diabetes mellitus. Nature 356 (6365), 162–164. 10.1038/356162a0 1545870

[B22] Guenat E. Seematter G. Philippe J. Temler E. Jequier E. Tappy L. (2000). Counterregulatory responses to hypoglycemia in patients with glucokinase gene mutations. Diabetes Metab. 26 (5), 377–384. 11119017

[B23] Guerin A. Nisenbaum R. Ray J. G. (2007). Use of maternal GHb concentration to estimate the risk of congenital anomalies in the offspring of women with prepregnancy diabetes. Diabetes Care 30 (7), 1920–1925. 10.2337/dc07-0278 17446531

[B24] Gupta R. K. Vatamaniuk M. Z. Lee C. S. Flaschen R. C. Fulmer J. T. Matschinsky F. M. (2005). The MODY1 gene HNF-4alpha regulates selected genes involved in insulin secretion. J. Clin. Invest. 115 (4), 1006–1015. 10.1172/JCI22365 15761495 PMC1059446

[B25] Hattersley A. T. Beards F. Ballantyne E. Appleton M. Harvey R. Ellard S. (1998). Mutations in the glucokinase gene of the fetus result in reduced birth weight. Nat. Genet. 19 (3), 268–270. 10.1038/953 9662401

[B26] Hughes A. E. Houghton J. A. L. Bunce B. Chakera A. J. Spyer G. Shepherd M. H. (2023). Bringing precision medicine to the management of pregnancy in women with glucokinase-MODY: a study of diagnostic accuracy and feasibility of non-invasive prenatal testing. Diabetologia 66 (11), 1997–2006. 10.1007/s00125-023-05982-9 37653058 PMC10542291

[B27] Kapoor R. R. Locke J. Colclough K. Wales J. Conn J. J. Hattersley A. T. (2008). Persistent hyperinsulinemic hypoglycemia and maturity-onset diabetes of the young due to heterozygous HNF4A mutations. Diabetes 57 (6), 1659–1663. 10.2337/db07-1657 18268044

[B28] Kyithar M. P. Bacon S. Pannu K. K. Rizvi S. R. Colclough K. Ellard S. (2011). Identification of HNF1A-MODY and HNF4A-MODY in Irish families: phenotypic characteristics and therapeutic implications. Diabetes Metab. 37 (6), 512–519. 10.1016/j.diabet.2011.04.002 21683639

[B29] Locke J. M. Dusatkova P. Colclough K. Hughes A. E. Dennis J. M. Shields B. (2022). Association of birthweight and penetrance of diabetes in individuals with HNF4A-MODY: a cohort study. Diabetologia 65 (1), 246–249. 10.1007/s00125-021-05581-6 34618178 PMC8660751

[B30] López Tinoco C. Sánchez L. B. Bacon S. Colclough K. Ng N. Wong E. (2021). Evaluation of pregnancy outcomes in women with GCK-MODY. Diabet. Med. 38 (6), e14488. 10.1111/dme.14488 33277730

[B31] Luni C. Marth J. D. Doyle F. J. 3rd (2012). Computational modeling of glucose transport in pancreatic β-cells identifies metabolic thresholds and therapeutic targets in diabetes. PLoS One 7 (12), e53130. 10.1371/journal.pone.0053130 23300881 PMC3531366

[B32] Lussier C. R. Brial F. Roy S. A. Langlois M. J. Verdu E. F. Rivard N. (2010). Loss of hepatocyte-nuclear-factor-1alpha impacts on adult mouse intestinal epithelial cell growth and cell lineages differentiation. PLoS One 5 (8), e12378. 10.1371/journal.pone.0012378 20808783 PMC2927538

[B33] McGlacken-Byrne S. M. Mohammad J. K. Conlon N. Gubaeva D. Siersbæk J. Schou A. J. (2022). Clinical and genetic heterogeneity of HNF4A/HNF1A mutations in a multicentre paediatric cohort with hyperinsulinaemic hypoglycaemia. Eur. J. Endocrinol. 186 (4), 417–427. 10.1530/EJE-21-0897 35089870

[B34] Menzel R. Kaisaki P. J. Rjasanowski I. Heinke P. Kerner W. Menzel S. (1998). A low renal threshold for glucose in diabetic patients with a mutation in the hepatocyte nuclear factor-1alpha (HNF-1alpha) gene. Diabet. Med. 15 (10), 816–820. 10.1002/(SICI)1096-9136(199810)15:10<816::AID-DIA714>3.0.CO;2-P 9796880

[B35] Menzel S. Yamagata K. Trabb J. B. Nerup J. Permutt M. A. Fajans S. S. (1995). Localization of MODY3 to a 5-cM region of human chromosome 12. Diabetes 44 (12), 1408–1413. 10.2337/diab.44.12.1408 7589847

[B36] Metzger B. E. Gabbe S. G. Persson B. Buchanan T. A. Catalano P. A. Damm P. (2010). International association of diabetes and pregnancy study groups recommendations on the diagnosis and classification of hyperglycemia in pregnancy. Diabetes Care 33 (3), 676–682. 10.2337/dc09-1848 20190296 PMC2827530

[B37] Mirshahi U. L. Colclough K. Wright C. F. Wood A. R. Beaumont R. N. Tyrrell J. (2022). Reduced penetrance of MODY-associated HNF1A/HNF4A variants but not GCK variants in clinically unselected cohorts. Am. J. Hum. Genet. 109 (11), 2018–2028. 10.1016/j.ajhg.2022.09.014 36257325 PMC9674944

[B38] Odom D. T. Zizlsperger N. Gordon D. B. Bell G. W. Rinaldi N. J. Murray H. L. (2004). Control of pancreas and liver gene expression by HNF transcription factors. Science 303 (5662), 1378–1381. 10.1126/science.1089769 14988562 PMC3012624

[B65] Østoft S. H. Bagger J. I. Hansen T. Pedersen O. Faber J. Halst J. J. (2014). Glucose-lowering effects and low risk of hypoglycemia in patients with maturity-onset diabetes of the young when treated with a GLP-1 receptor agonist: a double-blind, randomized, crossover trial. Diabetes Care 37 (7), 1797–1805. 24929431 10.2337/dc13-3007

[B39] Pearson E. R. Boj S. F. Steele A. M. Barrett T. Stals K. Shield J. P. (2007). Macrosomia and hyperinsulinaemic hypoglycaemia in patients with heterozygous mutations in the HNF4A gene. PLoS Med. 4 (4), e118. 10.1371/journal.pmed.0040118 17407387 PMC1845156

[B40] Pearson E. R. Starkey B. J. Powell R. J. Gribble F. M. Clark P. M. Hattersley A. T. (2003). Genetic cause of hyperglycaemia and response to treatment in diabetes. Lancet 362 (9392), 1275–1281. 10.1016/S0140-6736(03)14571-0 14575972

[B41] Pontoglio M. Prié D. Cheret C. Doyen A. Leroy C. Froguel P. (2000). HNF1alpha controls renal glucose reabsorption in mouse and man. EMBO Rep. 1 (4), 359–365. 10.1093/embo-reports/kvd071 11269503 PMC1083745

[B42] Ren Q. Wang Z. Yang W. Han X. Ji L. (2023). Maternal and infant outcomes in GCK-MODY complicated by pregnancy. J. Clin. Endocrinol. Metabolism 108 (10), 2739–2746. 10.1210/clinem/dgad188 37011183

[B43] Reznik Y. Dao T. Coutant R. Chiche L. Jeannot E. Clauin S. (2004). Hepatocyte nuclear factor-1 alpha gene inactivation: cosegregation between liver adenomatosis and diabetes phenotypes in two maturity-onset diabetes of the young (MODY)3 families. J. Clin. Endocrinol. Metab. 89 (3), 1476–1480. 10.1210/jc.2003-031552 15001650

[B44] Rowan J. A. Hague W. M. Gao W. Battin M. R. Moore M. P. MiG Trial Investigators (2008). Metformin versus insulin for the treatment of gestational diabetes. N. Engl. J. Med. 358 (19), 2003–2015. 10.1056/NEJMoa0707193 18463376

[B45] Shepherd M. Brook A. J. Chakera A. J. Hattersley A. T. (2017). Management of sulfonylurea-treated monogenic diabetes in pregnancy: implications of placental glibenclamide transfer. Diabet. Med. 34 (10), 1332–1339. 10.1111/dme.13388 28556992 PMC5612398

[B46] Shepherd M. H. Shields B. M. Hudson M. Pearson E. R. Hyde C. Ellard S. (2018). A UK nationwide prospective study of treatment change in MODY: genetic subtype and clinical characteristics predict optimal glycaemic control after discontinuing insulin and metformin. Diabetologia 61 (12), 2520–2527. 10.1007/s00125-018-4728-6 30229274 PMC6223847

[B47] Shields B. M. Hicks S. Shepherd M. H. Colclough K. Hattersley A. T. Ellard S. (2010). Maturity-onset diabetes of the young (MODY): how many cases are we missing? Diabetologia 53 (12), 2504–2508. 10.1007/s00125-010-1799-4 20499044

[B48] Spyer G. Macleod K. M. Shepherd M. Ellard S. Hattersley A. T. (2009). Pregnancy outcome in patients with raised blood glucose due to a heterozygous glucokinase gene mutation. Diabet. Med. 26 (1), 14–18. 10.1111/j.1464-5491.2008.02622.x 19125755

[B49] Stanescu D. E. Hughes N. Kaplan B. Stanley C. A. De León D. D. (2012). Novel presentations of congenital hyperinsulinism due to mutations in the MODY genes: HNF1A and HNF4A. J. Clin. Endocrinol. Metab. 97 (10), E2026–E2030. 10.1210/jc.2012-1356 22802087 PMC3674296

[B51] Steele A. M. Shields B. M. Wensley K. J. Colclough K. Ellard S. Hattersley A. T. (2014). Prevalence of vascular complications among patients with glucokinase mutations and prolonged, mild hyperglycemia. Jama 311 (3), 279–286. 10.1001/jama.2013.283980 24430320

[B52] Steele A. M. Wensley K. J. Ellard S. Murphy R. Shepherd M. Colclough K. (2013). Use of HbA1c in the identification of patients with hyperglycaemia caused by a glucokinase mutation: observational case control studies. PloS one 8 (6), e65326. 10.1371/journal.pone.0065326 23799006 PMC3683003

[B53] Stride A. Shepherd M. Frayling T. M. Bulman M. P. Ellard S. Hattersley A. T. (2002). Intrauterine hyperglycemia is associated with an earlier diagnosis of diabetes in HNF-1alpha gene mutation carriers. Diabetes Care 25 (12), 2287–2291. 10.2337/diacare.25.12.2287 12453975

[B54] Stride A. Shields B. Gill-Carey O. Chakera A. J. Colclough K. Ellard S. (2014). Cross-sectional and longitudinal studies suggest pharmacological treatment used in patients with glucokinase mutations does not alter glycaemia. Diabetologia 57 (1), 54–56. 10.1007/s00125-013-3075-x 24092492 PMC3855531

[B55] Timsit J. Ciangura C. Dubois-Laforgue D. Saint-Martin C. Bellanne-Chantelot C. (2021). Pregnancy in women with monogenic diabetes due to pathogenic variants of the glucokinase gene: lessons and challenges. Front. Endocrinol. (Lausanne) 12, 802423. 10.3389/fendo.2021.802423 35069449 PMC8766338

[B56] Towner D. Kjos S. L. Leung B. Montoro M. M. Xiang A. Mestman J. H. (1995). Congenital malformations in pregnancies complicated by NIDDM. Diabetes Care 18 (11), 1446–1451. 10.2337/diacare.18.11.1446 8722068

[B57] Tung J. Y. Boodhansingh K. Stanley C. A. De León D. D. (2018). Clinical heterogeneity of hyperinsulinism due to HNF1A and HNF4A mutations. Pediatr. Diabetes 19 (5), 910–916. 10.1111/pedi.12655 29493090 PMC6030428

[B58] Vaxillaire M. Boccio V. Philippi A. Vigouroux C. Terwilliger J. Passa P. (1995). A gene for maturity onset diabetes of the young (MODY) maps to chromosome 12q. Nat. Genet. 9 (4), 418–423. 10.1038/ng0495-418 7795649

[B59] Verhoef T. I. Hill M. Drury S. Mason S. Jenkins L. Morris S. (2016). Non-invasive prenatal diagnosis (NIPD) for single gene disorders: cost analysis of NIPD and invasive testing pathways. Prenat. Diagn 36 (7), 636–642. 10.1002/pd.4832 27107169 PMC6680142

[B60] Weng J. Ekelund M. Lehto M. Li H. Ekberg G. Frid A. (2002). Screening for MODY mutations, GAD antibodies, and type 1 diabetes--associated HLA genotypes in women with gestational diabetes mellitus. Diabetes Care 25 (1), 68–71. 10.2337/diacare.25.1.68 11772903

[B61] Yamagata K. Furuta H. Oda N. Kaisaki P. J. Menzel S. Cox N. J. (1996b). Mutations in the hepatocyte nuclear factor-4alpha gene in maturity-onset diabetes of the young (MODY1). Nature 384 (6608), 458–460. 10.1038/384458a0 8945471

[B62] Yamagata K. Oda N. Kaisaki P. J. Menzel S. Furuta H. Vaxillaire M. (1996a). Mutations in the hepatocyte nuclear factor-1alpha gene in maturity-onset diabetes of the young (MODY3). Nature 384 (6608), 455–458. 10.1038/384455a0 8945470

[B63] Zurawek M. Wender-Ozegowska E. Januszkiewicz-Lewandowska D. Zawiejska A. Nowak J. (2007). GCK and HNF1alpha mutations and polymorphisms in Polish women with gestational diabetes. Diabetes Res. Clin. Pract. 76 (1), 157–158. 10.1016/j.diabres.2006.08.001 16963153

